# An Anti-Inflammatory Composition of *Boswellia serrata* Resin Extracts Alleviates Pain and Protects Cartilage in Monoiodoacetate-Induced Osteoarthritis in Rats

**DOI:** 10.1155/2020/7381625

**Published:** 2020-05-21

**Authors:** Venkata Krishnaraju Alluri, Sreenath Kundimi, Krishanu Sengupta, Trimurtulu Golakoti, Eswar Kumar Kilari

**Affiliations:** ^1^Laila Nutraceuticals R&D Center, Vijayawada, Andhra Pradesh, India; ^2^AU College of Pharmaceutical Sciences, Andhra University, Visakhapatnam, Andhra Pradesh, India

## Abstract

The boswellic acids, the active compounds in *Boswellia serrata* gum resin extract, are potent anti-inflammatory agents and are specific nonredox inhibitors of 5-Lipoxygenase (5-LOX). Here, we present the anti-osteoarthritis (OA) efficacy of LI13019F1 (also known as Serratrin®), a unique composition containing the acidic and nonacidic fractions of *B. serrata* gum resin. This composition strongly inhibited 5-LOX activity with the half-maximal inhibitory concentration (IC_50_) of 43.35 ± 4.90 *μ*g/mL. Also, LI13019F1 strongly inhibited the leukotriene B_4_ (IC_50_, 7.80 ± 2.40 *μ*g/mL) and prostaglandin E_2_ (IC_50_, 6.19 ± 0.52 *μ*g/mL) productions in human blood-derived cells. Besides, LI13019F1 reduced TNF-*α* production with the IC_50_ of 12.38 ± 0.423 *μ*g/mL. On average, 1, 2.5, and 5 *μ*g/mL doses of LI13019F1 protected 34.62, 47.66, and 62.29% SW1353 human chondrosarcoma cells from IL-1*β* induced SOX-9 depletion, respectively. Further, a 28-day preclinical proof-of-concept study evaluated the pain relief efficacy of LI13019F1 in monoiodoacetate- (MIA-) induced *Sprague-Dawley* rats. At the end of the study, 150 and 300 mg/kg doses of LI13019F1 supplemented rats showed significant improvements (55.17 ± 5.81 g (*p* < 0.05), and 66.22 ± 6.30 g (*p* < 0.05), respectively, vs. MIA: 31.22 ± 7.15 g) in body-weight-bearing capacities. Concurrently, LI13019F1-150 and LI13019F1-300 rats substantially (*p* < 0.05) increased the threshold of pain sensitivity to pressure (26.98 ± 2.36 and 28.06 ± 2.72-gram force, respectively; vs. 18.63 ± 5.82 in MIA) and increased (*p* < 0.05) the latent time to withdraw the paw after a thermal stimulus (23.61 ± 2.73 and 28.18 ± 1.90 sec, respectively; vs. 16.56 ± 1.22 sec. in MIA). Besides, the histological observations on Safranin-O green stained articular cartilage revealed that LI13019F1 also prevented the MIA-induced structural damage of the cartilage and reduced the loss of the extracellular matrix (ECM) components in the experimental rats. In conclusion, the present observations suggest that LI13019F1, a new composition of *B. serrata* gum resin extracts, reduces pain and protects articular cartilage from the damaging action of MIA in a rodent model.

## 1. Introduction

Osteoarthritis (OA) is a debilitating disease, which primarily affects the hips and knees, the body-weight-bearing joints. Breakdown of the extracellular matrix of articular cartilage by the proinflammatory cytokine-induced tissue proteinases is the hallmark feature of the pathophysiology of OA [1]. The clinical presentations of OA are pain and degenerative changes in the tissues surrounding the affected joints [2]. Globally, OA of hip and knee is the 11^th^ highest contributor to the disability with enormous economic burden [3]. Some of the important factors that induce the progression of OA are chronic inflammation and gradual structural changes/structural remodeling within the joint tissues [4].

In progressive OA, gradual destruction of the structural integrity of the articular cartilage is the major pathophysiological basis of chronic pain. Pain is the primary clinical symptom of OA, and pain relief is the most important and priority need in OA management. The conventional nonsteroidal anti-inflammatory drugs (NSAIDs) and cyclooxygenase-2 (COX-2) inhibitors are the primary choices for symptomatic relief of inflammation and pain in OA [5, 6]. To minimize the side effects of NSAIDs on the gastrointestinal tract and platelet function, a novel strategy of combined inhibition of 5-LOX/COX has developed, and this dual inhibition strategy has shown great potential in OA pain management with improved tolerability [7, 8]. Besides, the use of a serotonin and norepinephrine reuptake inhibitor (Duloxetine) [9] or a transient vanilloid receptor 1 (TRPV-1) antagonist [10] has shown pain relief efficacy in OA. However, there are several approaches with established as well as emerging pain relief strategies in OA pain management but the therapeutic or preventive measures to protect or slow down the cartilage destruction process in progressive OA are unavailable [11]. We assume from a consumer's perspective that a product with a combined efficacy of pain relief and protection from articular cartilage damage might be the most attractive strategy in progressive OA management.

Gum resin extracts of *Boswellia serrata* or Indian Frankincense have been traditionally used in folk medicine for centuries. They have gained popularity among consumers to treat various chronic inflammatory conditions, namely, inflammatory bowel disease, asthma, allergies, arthritis, including osteoarthritis, and pain [12–16]. The gum resin of *Boswellia serrata* contains monoterpenes, diterpenes, triterpenes, tetracyclic triterpene acids, and pentacyclic triterpene acids, called boswellic acids (BAs). Early studies claimed that six major boswellic acids, namely, keto-*β*-boswellic acid (KBA), 3-O-acetyl-11-keto-*β*-boswellic acid (AKBA), *α*-boswellic acid (*α*-BA), *β*-boswellic acid (*β*-BA), 3-O-acetyl-*α*-boswellic acid (*α*-ABA), and 3-O-acetyl-*β*-boswellic acid (*β*-ABA), were responsible for the anti-inflammatory activities of the Boswellia gum resin [17]. These BAs exist in either *α*-configuration (geminal methyl groups at C-20) or *β*-configuration (vicinal methyl groups at C-19/C-20). The other structural characteristic features include the presence of a carbonyl moiety at C-11 in 11-keto-BAs and an acetyl moiety on the C-3 OH group in 3-O-acetyl-BAs. Besides, a carboxyl group is present in all the six BAs at C-24 [18, 19]. The Structure-Activity Relationship (SAR) studies on the BAs suggested that the carboxylic group and the ll-keto-group were essential for 5-lipoxygenase inhibition, and the acetyl-group on position C-3 OH had a moderate influence on the enzyme inhibition. Also, the Boswellia acids with *β*-configuration showed relatively better efficacy compared to the corresponding *α*-isomer [18, 19]. AKBA has optimum structural features, and in agreement, it showed the most potent 5-lipoxygenase inhibitory activity, which was closely followed by KBA. The BAs lacking the 11-keto function showed relatively weak enzyme-inhibitory activities [20]. AKBA and 11-keto-*β*-boswellic acid (KBA) also inhibit nuclear factor kappa B (NF-*κ*B), a nuclear factor that regulates the proinflammatory cytokine cascade, including TNF-*α* and IL-1*β* [21, 22]. A series of randomized, placebo-controlled clinical studies have established that various standardized preparations of *B. serrata* gum resin extracts are effective and safe alternative interventions for the management of OA pain [13–15, 23–25].

Here, we present a novel composition, LI13019F1 (also known as Serratrin®), containing the acidic and nonacidic fractions of *B. serrata* gum resin, standardized to six major BAs. Based on the assumptions, our primary focus of the study was to explore whether this composition could relieve pain and protects the articular cartilage in OA. In the present study, we tested whether LI13019F1 could block the production of 5-LOX and COX pathway derived inflammatory modulators and protect the chondrocytes from the damaging action of inflammatory cytokines in various cellular models. Further, a proof-of-concept study also evaluated the ability of LI13019F1 in relieving pain and cartilage protection in the MIA-induced OA model of *Sprague-Dawley* rats.

## 2. Materials and Methods

### 2.1. Study Material

**Figure 1:**
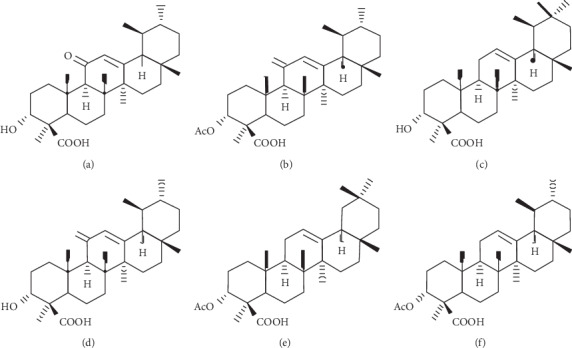
The major active boswellic acids present in LI13019F1. (a) 11-keto-*β*-boswellic acid (KBA), (b) 3-O-acetyl-11-keto-*β*-boswellic acid (AKBA), (c) *α*-boswellic acid (*α*-BA), (d) *β*-boswellic acid (*β*-BA), (e) 3-O-acetyl-*α*-boswellic acid (*α*-ABA), and (f) 3-O-acetyl-*β*-boswellic acid (*β*-ABA).

LI13019F1 (Serratrin®) is a composition of acidic and nonacidic fractions derived from an aqueous ethanol extract of *B. serrata* gum resin. To maintain the quality and batch-to-batch consistency, LI13019F1 was standardized to contain at least 30% of total BAs with not less than 5% Keto BAs. The major active boswellic acids present in LI13019F1 are 11-keto-*β*-boswellic acid (KBA; a), 3-O-acetyl-11-keto-*β*-boswellic acid (AKBA; b), *α*-boswellic acid (*α*-BA; c), *β*-boswellic acid (*β*-BA; d), 3-O-acetyl-*α*-boswellic acid (*α*-ABA; e), and 3-O-acetyl-*β*-boswellic acid (*β*-ABA; f) as depicted in Figure 1. In the standardization process, the concentrations of these six compounds in LI13019F1 were analyzed using pure phytochemical reference markers on high-performance liquid chromatography (HPLC) system supported with Empower™ 3 software (Waters Corporation, Milford, MA). The phytochemical markers were purchased from Sigma Chemical Co. (St. Louis, MO). The chromatographic separation was performed using Kinetex C18 2.6 *μ*m (100 × 4.6 mm) column (Phenomenex, Torrance, CA) and a gradient elution system consisting of solvent A (0.1% *v*/*v* orthophosphoric acid in water) and solvent B (acetonitrile and methanol at 45 : 55 *v*/*v*. The detailed analytical method and a typical HPLC chromatogram along with the plant raw material collection, extraction process, and the manufacturing process of LI13019F1 have been described earlier [26].

### 2.2. *In Vitro* Studies

#### 2.2.1. Human Blood Cells Isolation

Human blood was collected from healthy volunteers (Protocol number: LN/AIP/01/17 approved by ASR Ethics Committee, ASR Academy of Medical Sciences, Eluru, Andhra Pradesh, India) from a peripheral vein with a syringe containing 2 mM EDTA. Blood cells were isolated by centrifugation at 120 *g* for 10 minutes and resuspended in RPMI medium containing 10% FBS and 2 mM EDTA. Thirty milliliters of the blood cell suspension was layered onto 15 mL of ficoll/Lymphoprep in a 50 mL falcon tube in the dark and centrifuged at 350 ×*g* for 30 min. Buffy coat containing peripheral blood mononuclear cells (PBMCs) was collected carefully in 25 mL of cold 1X phosphate-buffered saline (PBS) and centrifuged at 150 *g* for 10 min. Residual RBC in the PBMC pellet was removed using ACK lysis buffer (Gibco, Thermo-Fisher Scientific, Waltham, MA). The RBC-free PBMCs were washed with fresh 1X PBS and resuspended in RPMI containing 10% FBS. The granulocytes were isolated using the ACK lysis buffer. The polymorphonuclear leukocytes (PMNs) were washed at 150 *g* for 10 min and resuspended in RPMI containing 1% newborn calf serum (Hyclone, GE Healthcare, Marlborough, MA).

#### 2.2.2. Cell Culture and Reagents

SW1353 human chondrosarcoma and THP-1 human monocytic cell lines were procured from American Type Culture Collection (ATCC, Manassas, VA). SW1353 and THP-1 cells were grown in Dulbecco's modified Eagle's medium (DMEM) (Sigma-Aldrich, St. Louis, MO) containing 10% fetal bovine serum (Thermo-Fisher Scientific, Waltham, MA). PromoCell (Heidelberg, Germany) was the source of human primary chondrocyte (HCH) cells and the chondrocyte growth medium. The experiments in the present study utilized the cultured chondrocytes of passage numbers between five and eight. The cells were passaged at about 80% confluence.

TNF-*α*, LTB*4* ELISA kits, and TACS XTT Cell Proliferation kit were procured from R&D Systems (Minneapolis, MN). PGE_2_ ELISA kit was purchased from Cayman Chemical (Ann Arbor, MI). Corning “V” bottom culture plates, dimethyl sulfoxide (DMSO), 1,9-Dimethylmethylene Blue zinc chloride double salt (DMMB), Lipopolysaccharides (LPS) from *Escherichia coli* O55: B5 (LPS), A23187, and remaining analytical grade reagents were purchased from Sigma-Aldrich (St. Louis, MO). Alexafluor® 647 tagged mouse anti-human SOX-9 antibody; True Nuclear Transcription factor buffer set was procured from BD Pharmingen^TM^ (Franklin Lakes, NJ).

#### 2.2.3. 5-Lipoxygenase Inhibition

5-LOX assay was carried out in a 96-well microplate utilizing ferric oxidation-xylenol orange (FOX) reagent as described earlier [27] with slight modifications. An increasing concentration of LI13019F1 (3.125–100 *μ*g/mL) was incubated with 5-LOX enzyme in each well of a 96-well microplate at 25°C for 5 minutes. A reagent mixture containing the 5-LOX enzyme in 50 mM Tris-HCl buffer (pH 7.4) with 0.2% DMSO ran in parallel as the vehicle control. The enzyme reaction started with the addition of 140 *μ*M linoleic acid in 50 mM Tris-HCl buffer, pH 7.4, and the reaction mixture was incubated at 25°C for 20 min in the dark. The assay was terminated by the addition of freshly prepared FOX reagent (30 mM sulfuric acid, 100 *μ*M xylenol orange, 100 *μ*M iron (II) sulfate in 9 : 1 methanol/water solution). After the color development at 25°C, the absorbance was read at 595 nm using a microplate reader (xMark^TM^, Bio-Rad, Hercules, CA).

#### 2.2.4. Leukotriene B_4_ Assay

An equal number of PMNs seeded in each well of a 96-well microplate was treated with different concentrations of LI13019F1. The vehicle control wells received 0.2% DMSO (*v*/*v*). The plate was incubated in a CO_2_ incubator at 37°C for 2 hr. The cells were induced with 10 µM A23187 for a further 10 min at 37°C in the presence of 5% CO_2_. An ELISA kit measured the LTB_4_ content in the cell-free culture supernatants (clarified at 270 g, 5 min), following the manufacturer's instructions (R&D Systems, Minneapolis, MN). The assay utilized the competitive antigen-antibody binding principle. Following the substrate reaction, the absorbance of the developed color was measured at 450 nm in a microplate reader (Spectramax 2e; Molecular Devices, San Jose, CA). The amount of LTB_4_ present in the supernatants was quantified, comparing the optical density values with a standard curve plotted against the known concentrations of the analyte.

#### 2.2.5. Prostaglandin E_2_ Assay

PGE_2_ inhibitory activity of LI13019F1 was evaluated in human PBMCs [28]. Briefly, an equal number of PBMCs were seeded in each well of a 96-well culture plate, and the cells were treated with different concentrations of LI13019F1 for 2 hr at 37°C in a CO_2_ incubator. Then, the cells were induced with 10 ng/mL LPS and incubated further for 4 hr. The cell culture wells received only 0.2% DMSO served as the vehicle control. The PGE_2_ was measured in the cell-free culture supernatant (clarified at 270 g, 5 min), using an ELISA kit. The PGE_2_ assay was performed following the manufacturer's instructions. Absorbance was measured at 412 nm in a microplate reader (Spectramax 2e; Molecular Devices, San Jose, CA).

#### 2.2.6. TNF-*α* Assay

An equal number of human PBMCs suspended in phenol red-free DMEM containing 1% FBS were plated in a 96-well flat-bottom culture plate. Cells were pretreated with different concentrations of LI13019F1 at 37°C for 2 hr in the presence of 5% CO_2_. After that, the cells were induced with 1 *μ*g/ml LPS and incubated further for 4 hr. The vehicle control culture wells received only 0.1% (*v*/*v*) DMSO in DMEM-1% FBS. TNF-*α* present in the cell-free culture supernatants (clarified at 270 *g*, 10 min) was quantified using a Human TNF-*α* ELISA kit (R&D Systems, Minneapolis, MN). The assay was performed following the manufacturer's instructions. Absorbance was measured at 450 nm in a microplate reader (Spectramax 2e; Molecular Devices, San Jose, CA). An increasing concentration of TNF-*α* was run in parallel to construct a standard curve for the quantitative measurement of TNF-*α* present in the culture supernatants.

#### 2.2.7. Chondrocyte Cell Proliferation Assay

LI13019F1 was evaluated for its ability to modulate the survival of IL-1*β*-induced HCH human primary chondrocytes. Briefly, an equal number of HCH cells were plated in each well of a 96-well cell culture plate and incubated overnight at 37°C in the presence of 5% CO_2_. The next day, the culture medium was replaced with fresh medium containing IL-1*β* (5 ng/mL) and allowed to grow for 72 hr in the presence or absence of increasing concentrations of LI13019F1. The vehicle control cultures received only 0.2% DMSO (*v*/*v*) containing growth medium. Following the treatment period, the culture wells were washed with serum-free and phenol red-free DMEM. Finally, an equal volume of XTT reagent working solution was added to each well and incubated in the dark at 37°C. The absorbance at 490 nm with a reference wavelength of 630 nm was determined using a microplate reader (Spectramax 2e; Molecular Devices, San Jose, CA). The absorbance values in the vehicle control wells were considered as 100 percent cell viability.

#### 2.2.8. SRY-Related High-Mobility Group-Box 9 (SOX-9) Assay

SOX-9 assay was performed using a BD FACSVerse flow cytometer (BD Biosciences, San Jose, CA). Briefly, an equal number of SW1353 cells in each well of a 96-well flat-bottom cell culture plate were preincubated with 5 ng/mL IL-1*β* for 1 hr in the presence or absence of various concentrations of LI13019F1 for further 3 hr at 37°C in a CO_2_ incubator. The cells in each well were resuspended and transferred to a “V”-bottom plate and stained with Alexafluor® 647 tagged mouse anti-human SOX-9 antibody following the intracellular staining protocol using True Nuclear Transcription Factor Buffer Set from BD Pharmingen™. The stained and unstained cells were incubated with only fluorescence tagged antibody and 0.2% DMSO, respectively. The cell suspensions were acquired on a BD FACSVerse flow cytometer and analyzed by BD FACS Suite software.

#### 2.2.9. Glycosaminoglycan (GAG) Assay

GAG staining with 1,9-dimethylmethlyene blue (DMMB) in chondrocytes was employed to evaluate whether LI13019F1 can modulate cartilage matrix production in IL-1*β*-induced HCH human primary chondrocytes [29]. Briefly, an equal number of HCH cells were seeded in each well of a 48-well cell culture plate. The cells were treated with 5 ng/mL of IL-1*β* in the presence or absence of an increasing concentration of LI13019F1 and incubated for 72 hr at 37°C in the presence of 5% CO_2_. Following the treatment period, the washed cells were lysed in cell lysis buffer, and an equal volume of cell lysates was reacted with the DMMB dye reagent (16 mg DMMB in 95 mL 0.1 M acetic acid containing 3.04 g glycine and 1.6 g NaCl). Immediately after the color development, the absorbance was read at 525 nm in a microplate reader (Spectramax 2e; Molecular Devices, San Jose, CA). Increasing concentrations of chondroitin sulfate A were run in parallel to construct a standard curve for the quantitative measurement of the ECM components present in the cell lysates.

### 2.3. *In Vivo* Study

#### 2.3.1. Animals

Specific pathogen-free female *Sprague-Dawley* rats, aged 8–12 weeks (195–229 g body weight), were purchased from Palamur Biosciences Private Limited, Hyderabad, India. Before the start of the experiment, the animals were acclimatized to the laboratory conditions (23 ± 2°C temperature and 40–70% relative humidity and 12 hr light/dark cycle) for seven days. They received a standard rodent pellet diet (Krishna Valley Agrotech, Pune, India) and mineral water *ad libitum*. The animal handling and the experimental procedures followed the guidelines of the Committee for the Purpose of Control and Supervision of Experiments on Animals (CPCSEA), India. The Institutional Animal Ethics Committee (IAEC) of Laila Impex, Vijayawada, India, approved the experimental protocol (Protocol no. LI/IAEC/LI190108).

#### 2.3.2. Oral Administration of LI13019F1 in MIA-Induced Sprague-Dawley Rats

Following the acclimatization period, on day 1 of the experiment, twenty-four rats received a single intra-articular injection of 1 mg MIA in 25 *μ*l of sterile normal saline into the right hind knee joint through patellar ligament under ketamine anesthesia. Animals in the control group (G1) received an equal volume of sterile normal saline (NS) into the right hind knee joint. On day 4, the MIA-induced animals were randomly allocated into four groups (*n* = 6) based on their body-weight-bearing capacity. The animals received a daily dose of either 0.5% Carboxymethylcellulose Sodium (CMC) (Group 2, G2) or CMC containing 75 mg/kg (Group 3, G3) or 150 mg/kg (Group 4, G4) or 300 mg/kg (Group 5, G5) of LI13019F1 through oral gavage from day 4 through the end of the study, i.e., day 28. In parallel, the NS-administered animals (G1) also received 0.5% CMC through oral gavage during the experiment.

#### 2.3.3. Assessment of Body-Weight-Bearing Capacity

After induction of OA, the body-weight-bearing capacities of the experimental rats on their right hind paws were measured on days 4, 7, 14, 21, and 28 using an Incapacitance Meter (IITC Life Science Inc., Woodland Hills, CA) [30] with slight modifications. Briefly, the animals were placed in a measuring chamber where the animal rested their hind paws on two separate sensor plates. The sensor plates measured the force exerted by the right-back legs. A mean of six consecutive readings was considered as the individual animal data. Following the instruction manual, the equipment was calibrated on the days of measurements using a one hundred-gram check weight.

#### 2.3.4. Thermal and Mechanical Paw Withdrawal Tests

The hyperalgesia or sensitivities to thermal and mechanical pain were tested using a Plantar Test Apparatus (Plantar Test, Ugo Basile, Italy) [30] and with an Electronic Von Frey instrument (Electronic Von Frey, Ugo Basile, Italy). These assessments were performed on days 5, 15, and 27 following the standard protocols. The rats were adapted with the test apparatus. Before MIA injection, the baseline latencies to the paw withdrawals on thermal or mechanical stimulations were measured. In the thermal sensitivity experiment, the rats received a radiant heat stimulus of approximately 30 IR on the plantar surface of the right hind paw. A cut-off time of 20 sec was maintained to avoid potential tissue damage. The result of each animal was expressed as the mean of three trials.

An Electronic Von Frey filament generated the mechanical stimulation to test pain sensitivity. The filament was pressed against the ventral side of the right hind paw of the experimental rats and recorded the force (gf) at the moment of paw withdrawal. Mechanical paw withdrawals were tested using the same study design as described for thermal withdrawal tests. The withdrawal threshold was expressed as mean gram force (gf) of three independent measurements conducted with an interval of 20 min.

#### 2.3.5. Histopathology

On day 29, following necropsy, the right hind knee joints of the experimental rats were collected. The joint tissues were fixed in 10% neutral buffered formalin for 48 hr and paraffin-embedded following the standard method [31]. Joints tissues were decalcified in Gooding and Stewart's decalcification fluid (20% (*v*/*v*) formic acid, 5% (*v*/*v*) formaldehyde) and processed in an automatic tissue processor (Leica ASP300S) for paraffin-embedding. The paraffin-embedded tissue blocks were sectioned at a thickness of 4 *μ*m using a manual rotary microtome (Microtome RTS2125, Leica Biosystems, Mumbai, India). The tissue sections were stained with Safranin-O green stain and examined under a microscope at 10X (Axio Scope A1, Carl Zeiss GmbH, Jena, Germany). A CCD camera (ProgRes C5, Genoptik, Jena, Germany) captured the bright-field images.

### 2.4. Statistical Analysis

The data was presented as mean ± SD. The data were analyzed using two-way ANOVA via Bonferroni's post hoc analysis utilizing GraphPad Prism software v5.01 (GraphPad Software, Inc., CA, USA). A *p* value of <0.05 was considered statistically significant.

## 3. Results

### 3.1. LI13019F1 Downregulates the Key Mediators of Inflammatory Pain

**Figure 2:**
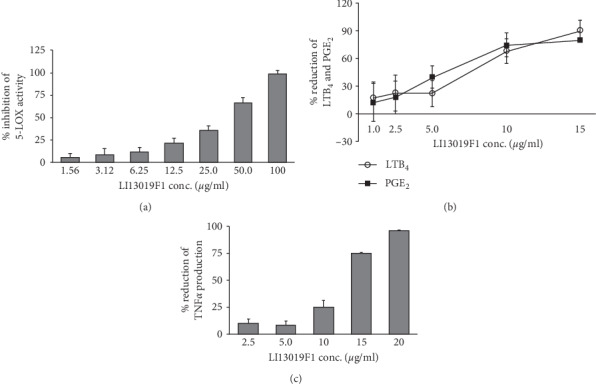
LI13019F1 downregulates inflammatory modulators *in vitro*. (a) The bar diagram presents a dose-dependent inhibition of 5-lipoxygenase enzyme activity by LI13019F1 in ferrous oxidation-xylenol orange (FOX) assay. (b) The line graphs show dose-dependent reductions of LTB_4_ and PGE_2_ productions in A23187-induced human polymorphonuclear neutrophils (PMNs) and LPS-induced human peripheral mononuclear cells (PBMCs), respectively. (c) The bar graph presents dose-dependent reductions of TNF-*α* production in LPS-induced human PBMC, as indicated. Data show mean ± SD; *n* = 3.

Figure 2(a) presents a dose-dependent inhibition of 5-LOX activity by LI13109F1 with an IC_50_ of 43.35 ± 4.90 *μ*g/mL. Also, LI13019F1 strongly inhibited the LTB_4_ production in A23187, a calcium ionophore-induced human blood-derived PMNs with an IC_50_ of 7.80 ± 2.40 *μ*g/mL (Figure 2(b)). LTB_4_ is an essential inflammatory leukotriene produced from arachidonic acid metabolism. Further, we also observed that LI13019F1 strongly inhibited PGE_2_ production (IC_50_, 6.19 ± 0.52 *μ*g/mL) in LPS-induced human PBMCs (Figure 2(b)). The cyclooxygenase (COX) pathway derived PGE_2_ is a potent mediator of inflammatory and neuropathic pain.

To examine the effects of LI13019F1 on inhibition of a major proinflammatory cytokine such as TNF-*α*, we utilized human PBMCs. We observed that LI13019F1 dose-dependently inhibited TNF-*α* production in LPS-stimulated PBMCs. The results demonstrate that the IC_50_ of LI13019F1 to inhibit TNF-*α* production is 12.38 ± 0.423 *μ*g/mL (Figure 2(c)).

### 3.2. LI13019F1 Protects Human Primary Chondrocytes from the Harmful Effects of IL-1*β*

**Figure 3:**
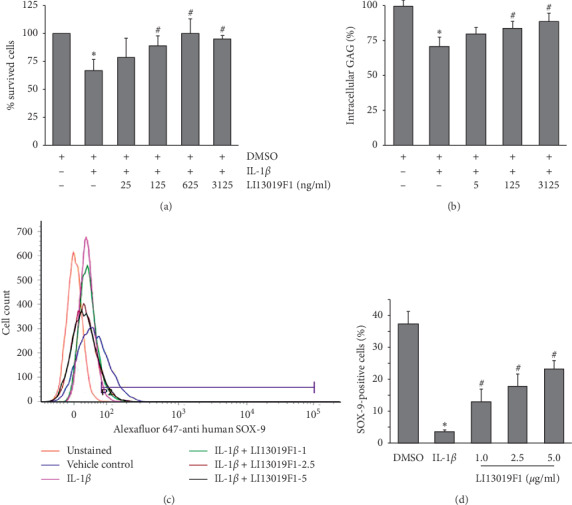
LI13019F1 mitigates the harmful effects of proinflammatory cytokines on chondrocytes. (a) The bar diagram shows the effect of an increasing concentration of LI13019F1 on human primary chondrocytes survivability in the presence of IL-1*β*. The absorbance values obtained in the vehicle control culture wells were considered as 100% cell viability. Each bar presents the data of mean ± SD, *n* = 4. (b) The bar diagram shows the modulation in intracellular glycosaminoglycan (GAG) content at various concentrations of LI13019F1-treated cells in the presence of IL-1*β*, as indicated. The amount of GAG present in the 0.2% DMSO-treated cells (vehicle control) was presented as 100 percent. Each bar shows a mean ± SD. *n* = 4. (c) & (d) the histogram and bar diagram show the efficacy of LI13019F1 in recovering the SOX-9-positive SW1353 human chondrosarcoma cells (in percentage) following the IL-1*β* induction. Each bar presents a mean ± SD of percent SOX-9-positive cells from three independent experiments. ^*∗*^ (vs. vehicle control) and # (vs. IL-1*β* treated) indicate significance (*p* < 0.05), using two-way ANOVA.

Proinflammatory cytokines induce apoptosis and reduce the viability of the articular chondrocytes in progressive OA [32]. In the present study, we intended to evaluate whether LI13019F1 can modulate the viability of human primary chondrocytes when cotreated with IL-1*β in vitro*. We performed an XTT incorporation-based cell proliferation assay to assess the effect of LI13019F1 on cell survivability in IL-1*β*-treated HCH human primary chondrocytes. In cell proliferation experiment, we observed that IL-1*β* treatment significantly (*p* < 0.05) reduced the chondrocyte viability by a mean of 32.66%, compared with the vehicle control cultures. At the same time, the LI13019F1-treated cultures showed remarkable recovery from the IL-1*β*-induced loss of cell viability. The mean ± SD of percent viable cells recorded in IL-1*β*-treated cells in the presence of 25, 125, 625, and 3125 ng/mL of LI13019F1 cultures are 79.65 ± 16.60%, 89.49 ± 8.57%, 100.15 ± 13.6%, and 95.34 ± 3.36%, respectively (Figure 3(a)). In comparison with the IL-1*β*-treated cultures, the number of viable cells in 125, 625, and 3125 ng/mL LI13019F1-treated cultures was significant.

Next, we evaluated the ability of LI13019F1 to improve the extracellular matrix (ECM) components in the IL-1*β*-induced chondrocytes. Glycosaminoglycans (GAGs) are the major backbone of the proteoglycans, the essential ECM components of the noncellular part of the articular cartilage. The chondrocytes synthesize GAG, and its content in the cartilage is proportional to the OA severity [33]. We measured the intracellular GAG content in the absence or presence of LI13019F1 in IL-1*β*-induced primary chondrocytes. Our observation shows that upon IL-1*β* treatment, the cellular GAG content is reduced by a mean of 28.88% from the vehicle-treated cells. An increasing concentration of LI13019F1 supplementation showed a gradual recovery of intracellular GAG in the primary chondrocytes. The intracellular GAG was increased to 79.5 ± 4.58%, 84.06 ± 4.99% (*p* < 0.05, vs. IL-1*β* treated), and 88.73 ± 6.01% (*p* < 0.05, vs. IL-1*β* treated) in 5, 125, and 3125 ng/ml LI13019F1-supplemented cultures in the presence IL-1*β*, respectively (Figure 3(b)).

Further, to explore the protective efficacy of LI13019F1, we tested the modulation of SOX-9 expression in IL-1*β*-induced SW1353 human chondrosarcoma cells. The transcription factor SOX-9 is the master regulator for chondrogenesis and essential for its homeostasis [34]. In our experiment, the flow cytometry analyses demonstrate that IL-1*β*-treated culture wells contained 3.60 ± 0.57% SOX-9-positive cells (in contrast, 37.39 ± 3.85% in the vehicle control culture). When the IL-1*β*-treated cells were coincubated with 1, 2.5, or 5 *μ*g/mL of LI13019F1, the population of the SOX-9 positive cells increased to 12.94 ± 4.01%, 17.82 ± 3.72%, and 23.29 ± 2.69%, respectively (Figures 3(c) and 3(d)). These increases were significant when compared with the IL-1*β*-treated culture. On average, 1, 2.5, and 5 *μ*g/ml doses of LI13019F1 protected 34.62, 47.66, and 62.29% cells, respectively, from IL-1*β*-induced SOX-9 depletion.

### 3.3. LI13019F1 Supplementation Improves Weight-Bearing Capacity in the Experimentally Induced Osteoarthritic Sprague-Dawley Rats

**Figure 4:**
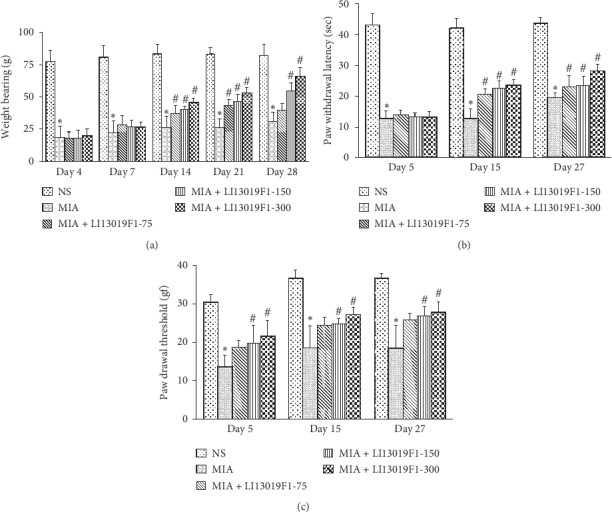
LI13019F1 alleviates pain and improves sensitivity to pain stimuli in MIA-induced *Sprague-Dawley* rats. (a) The graphical presentation shows gradual improvements in body-weight-bearing (g) capabilities of the LI13019F1-supplemented rats. The bar graphs in panels (b) and (c) show reductions in pain sensitivities to the thermal (in sec) and mechanical (in gram force) stimuli tested in the thermal probe test and von Frey test, respectively. The descriptions of the experimental groups are in the materials and methods section. The bars present the data mean ± SD; *n* = 6. ^*∗*^ (vs. NS group) and # (vs. MIA group) indicate significance (*p* < 0.05), using two-way ANOVA. NS, normal saline.

We assessed the weight-bearing capabilities of all animals on days 4, 7, 14, 21, and 28. Intra-articular administration of MIA produced a typical OA symptom like knee pain, and that was evident as a significant reduction of the body-weight-bearing capability of the MIA-induced rats (G2), compared with the NS-rats (G1) (Figure 4(a)). The LI13019F1-supplemented rats (G3-G5) showed gradual improvements in weight-bearing capacities through the experiment. Interestingly, significant increases in weight-bearing ability started from as early as day 14. The LI13019F1-150 and LI13019F1-300 rats showed significantly (*p* < 0.05) improved weight-bearing function on days 14, 21, and 28 when compared with the MIA rats (Figure 4(a)). On days 14 and 21, the improvements in the LI13019F1-75 rats were significant (*p* < 0.05, vs. MIA rats), while the change on day 28 (vs. MIA rats) was not significant (Figure 4(a)).

### 3.4. LI13019F1 Supplementation Improves Sensitivity to Thermal and Mechanical Stimuli in MIA-Induced Rats

We assessed the pain sensitivity to the thermal stimulus on days 5, 15, and 27 of the experiment using a Plantar Test Apparatus. The MIA rats (G2) exhibited significantly reduced (*p* < 0.05) hind paw withdrawal latency than the control animals (G1) from day 5 through the end of the study. LI13019F1-supplemented rats showed significant improvements in the latency of the paw withdrawal, in comparison with the MIA rats (G2), starting from day 15 of the experiment (Figure 4(b)). Daily doses of LI13019F1 at 75, 150, and 300 mg/kg resulted in significant and dose-dependent improvements in paw withdrawal latency (sec) (*p* < 0.05) compared to MIA-induced rats on day 15. At the final evaluation on day 27, LI13019F1-300 rats (G5) showed significant improvement (*p* < 0.05) compared to MIA control (G2), as represented in Figure 4(b).

Mechanical hyperalgesia (allodynia) was evaluated on days 5, 15, and 27 of the experiment. MIA group (G2) showed a significantly reduced (*p* < 0.05) paw withdrawal threshold compared with the normal-saline-injected animals (G1) through the study duration. The mid-dose (G4) and high dose (G5) of LI13019F1-supplemented rats showed gradual improvements in pain sensitivity in comparison with the MIA-induced rats (G2) (Figure 4(c)). These improvements were significant (*p* < 0.05), compared with G2, starting from day 5 through the end of the study. The LI13019F1-75 rats (G3) also showed improvement in the pain threshold compared with the MIA rats, but these changes were not significant (Figure 4(c)).

### 3.5. LI13019F1 Supplementation Improves Osteoarthritic Changes in MIA Rats

**Figure 5:**
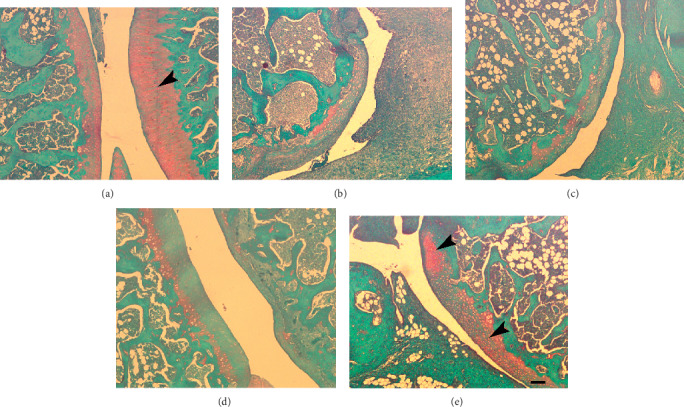
LI13019F1 protects cartilage from the damaging action of MIA in *Sprague-Dawley* rats. Representative photomicrographs show Safranin-O green stained articular cartilage tissue sections of the LI13019F1-supplemented rats, as indicated. The panels (a), (b), (c), (d), and (e) represent the experimental groups as described in the materials and methods, i.e., normal saline, MIA-induced in the absence or presence of 75, 150, and 300 mg/kg LI13019F1 rats, respectively. The arrowheads show the presence of extracellular matrix components (red stain) and cellular structures (chondrocytes) in the articular surface of the joints. The scale bar measures 100 *μ*m.

Figure 5 presents representative photomicrographs of Safranin-O green stained articular cartilage of the knee joints of the experimental rats.  Figure 5(a) shows a typical presentation of a healthy cartilage structure with an abundant number of chondrocytes and profuse red stain in the NS group (G1). In the MIA-induced (G2) group, cartilage erosion and the presence of a few numbers of chondrocytes along with a faint red stain are visible (Figure 5(b)). The LI13019F1-150 (G4) and LI13019F1-300 (G5) rats show improved cartilage structure with an increasing population of chondrocytes and red stain in Figures 5(d) and 5(e), respectively. A deeper red stain indicates the presence of increased ECM components in the articular cartilage in the LI13019F1-supplemented groups.

## 4. Discussion

Here, we present a novel anti-inflammatory composition of the acidic and nonacidic fractions of *B. serrata* gum resin extracts, which alleviates osteoarthritis pain and protects from articular cartilage damage in an experimental rodent model. Various preparations of the gum resin extracts of this plant are known to be anti-inflammatory [35–37], and these preparations alleviated the clinical symptoms of a variety of inflammatory ailments, including OA [13, 14]. The *B. serrata* gum resin extract-derived formulations are claimed to inhibit the 5-lipoxygenase pathway for their anti-inflammatory activities in the management of OA [15, 16, 38]. However, the COX inhibitors, e.g., NSAIDs, are the choice of drugs to manage the major clinical symptom of OA, i.e., pain [5, 6], but the side effects limit their long-term use [8]. Therefore, to improve the benefit/ risk ratio in pain management of OA, a dual inhibition strategy (COX and LOX combined inhibition) has shown a great promise [4, 39, 40]. To the best of our knowledge, this is the first report on a *Boswellia* resin extract composition that coinhibits the productions of LOX and COX pathway-derived inflammatory mediators. In the present study, we systematically evaluated the efficacy of LI13019F1 in blocking the productions of the inflammatory pain mediators in cell-based models followed by a preclinical proof-of-concept study to demonstrate its pain relief efficacy in MIA-induced OA rat model. Besides, we have also shown the ability of LI13019F1 to protect the articular cartilage from the damaging action of MIA in the experimental animals.

MIA-induced OA in rodents is a widely accepted experimental model. This model resembles the phenotypes of articular cartilage degeneration in OA of humans. MIA inhibits glyceraldehyde-3-phosphatase dehydrogenase, gradually destroys cartilage, and generates pain like symptoms [30, 41]. The observations from our *in vitro* experiments suggest that LI13019F1 is a promising candidate to alleviate pain through combined inhibition of COX and LOX pathways of the arachidonic acid metabolism. The preclinical efficacy study data substantiates the hypothesis drawn from the *in vitro* observations and establishes that LI13019F1 is a potential analgesic composition to improve the pain sensitivity in OA. The pain-behavior studies in rodent models routinely evaluate the stimulus-independent and stimulus-dependent nociception through their surrogate measures such as body-weight bearing and pain sensitivity to thermal or mechanical stimulus, respectively [42].

The proinflammatory cytokines such as TNF-*α* and IL-1*β* play a crucial role in the onset and progression of OA. These cytokines induce apoptosis and block the synthesis of ECM components in the chondrocytes [43]. Also, in chondrocyte homeostasis, the transcription factor SOX-9 plays an essential role in activating the marker genes for chondrogenesis and extracellular matrix productions [34]. Proinflammatory cytokines downregulate SOX-9 protein expression via an NF-*κ*B-regulated signaling pathway [44, 45] The present *in vitro* data on TNF-*α* production inhibition suggests that LI13019F1 is an anti-inflammatory Boswellia composition. In parallel, the ability of LI13019F1 in protecting the cells from the harmful effects of IL-1*β* on SOX-9 depletion, cell survival, and intracellular glycosaminoglycan (GAG) content is encouraging. GAG is one of the major soluble extracellular matrix (ECM) components, synthesized in the chondrocytes. Its synthesis decreases when the intracellular SOX-9 is downregulated through retinoid receptor RAR*α* activation in an inflammatory state [46]. However, these parallel observations from the *in vitro* experiments collectively suggest a promising role of LI13019F1 in protecting the cartilage against the damaging action of inflammatory modulators in progressive OA. Further, the histology study data of the Safranin-O green stained joints support the *in vitro* observations and suggest a potential chondroprotective role of LI13019F1.

In the present study, we have not tested the specificity of LI13019F1 on COX inhibition. Also, the further exploration of the specific mechanism of action of LI13019F1 in pain alleviation and its involvement in SOX-9 signaling in cartilage protection would be exciting. In the present *in vivo* efficacy study, we did not measure whether LI13019F1 supplementation yielded benefit in other cartilage matrix substances such as collagen-II and proteoglycans or in improving the anabolic/catabolic balance in the MIA-induced OA rats. The preclinical study presented here is a proof-of-concept *in vivo* efficacy study to demonstrate the effect of this botanical composition on cartilage protection. We plan to address the unanswered queries in our future studies. Importantly, the oral consumption of LI13019F1 does not possess safety concerns. Recently, in a 28-day subacute oral safety study and genotoxicity studies, we have established a broad spectrum preclinical safety of LI13019F1 [25]. Overall, the present observations are encouraging and provide strong evidence that LI13019F1 is a potential candidate to mitigate pain and provide cartilage protection in human subjects with OA.

## 5. Conclusions

The present *in vitro* and *in vivo* data demonstrate that the standardized composition of *Boswellia serrata* gum resin extracts, LI13019F1 (Serratrin®), relieves pain through combined inhibition of cyclooxygenase and 5-lipoxygenase pathways. Besides, the *in vitro* observations show that LI13019F1 mitigates the harmful effect of the proinflammatory cytokine on an essential transcription factor SOX-9, which maintains chondrocyte homeostasis and its survival. Further, in support, the *in vivo* observation shows evidence that LI13019F1 can provide cartilage protection in an experimentally induced osteoarthritis in *Sprague-Dawley* rats. Together, these data suggest that LI13019F1 would be a novel candidate for managing pain with an additional benefit of cartilage protection in progressive OA in humans.

## Data Availability

Data and publication materials are available upon request.
